# Individualized PEEP can improve both pulmonary hemodynamics and lung function in acute lung injury

**DOI:** 10.1186/s13054-025-05325-7

**Published:** 2025-03-10

**Authors:** Mayson L. A. Sousa, Luca S. Menga, Annia Schreiber, Mattia Docci, Fernando Vieira, Bhushan H. Katira, Mariangela Pellegrini, Sebastian Dubo, Ghislaine Douflé, Eduardo L. V. Costa, Martin Post, Marcelo B. P. Amato, Laurent Brochard

**Affiliations:** 1 Keenan Centre for Biomedical Research, Li Ka Shing Knowledge Institute, St. Michael’s Hospital, Unity Health Toronto, Toronto, Canada; 2 Interdepartmental Division of Critical Care Medicine, University of Toronto, Toronto, Canada; 3 Translational Medicine Program, Research Institute, Hospital for Sick Children, Toronto, Canada; 4 Department of Respiratory Therapy, Rady Faculty of Health Sciences, University of Manitoba, 771 McDermot Avenue, Room 338, Winnipeg, Manitoba R3M 1S1 Canada; 5 Pulmonary Division, University of Sao Paulo, Sao Paulo, Brazil; 6 Pediatric Critical Care Medicine, Department of Pediatrics, Washington University in St Louis, St Louis, USA; 7 Anesthesiology and Intensive Care Medicine, Department of Surgical Sciences, Uppsala University, Uppsala, Sweden; 8 Hedenstierna Laboratory, Department of Surgical Sciences, Uppsala University, Uppsala, Sweden; 9 Department of Physiotherapy, Universidad de Concepción, Concepción, Chile; 10 Institute of Health Policy, Management, and Evaluation, University of Toronto, Toronto, Canada; 11 Department of Anesthesia and Pain Management, Toronto General Hospital, University Health Network, Toronto, Canada; 12 Department of Physiology, University of Toronto, Toronto, Canada

**Keywords:** Positive end-expiratory pressure, Pulmonary hemodynamics, Pulmonary vascular resistance, Mechanical ventilation, Acute lung injury

## Abstract

**Rationale:**

There are several approaches to select the optimal positive end-expiratory pressure (PEEP), resulting in different PEEP levels. The impact of different PEEP settings may extend beyond respiratory mechanics, affecting pulmonary hemodynamics.

**Objectives:**

To compare PEEP levels obtained with three titration strategies—(i) highest respiratory system compliance (C_RS_), (ii) electrical impedance tomography (EIT) crossing point; (iii) positive end-expiratory transpulmonary pressure (P_L_)—in terms of regional respiratory mechanics and pulmonary hemodynamics.

**Methods:**

Experimental studies in two porcine models of acute lung injury: (I) bilateral injury induced in both lungs, generating a highly recruitable model (n = 37); (II) asymmetrical injury, generating a poorly recruitable model (n = 13). In all experiments, a decremental PEEP titration was performed monitoring P_L_, EIT (collapse, overdistention, and regional ventilation), respiratory mechanics, and pulmonary and systemic hemodynamics.

**Measurements and main results:**

PEEP titration methods resulted in different levels of median optimal PEEP in bilateral lung injury: 14(12–14) cmH_2_O for C_RS_, 11(10–12) cmH_2_O for EIT, and 8(8–10) cmH_2_O for P_L_, *p* < 0.001. Differences were less pronounced in asymmetrical lung injury. PEEP had a quadratic U-shape relationship with pulmonary artery pressure (R^2^ = 0.94, *p* < 0.001), right-ventricular systolic transmural pressure, and pulmonary vascular resistance. Minimum values of pulmonary vascular resistance were found around individualized PEEP, when ventilation distribution and pulmonary circulation were simultaneously optimized.

**Conclusions:**

In porcine models of acute lung injury with variable lung recruitability, both low and high levels of PEEP can impair pulmonary hemodynamics. Optimized ventilation and hemodynamics can be obtained simultaneously at PEEP levels individualized based on respiratory mechanics, especially by EIT and esophageal pressure.

**Supplementary Information:**

The online version contains supplementary material available at 10.1186/s13054-025-05325-7.

## Introduction

Setting positive end-expiratory pressure (PEEP) is a crucial aspect of mechanical ventilation and remains a challenge in clinical practice [1–5]. The optimal strategy for PEEP titration remains a topic of debate [6–8], and there is no universally accepted method. The PEEP-FiO_2_ table strategy [9] has been widely used because of its easy applicability, but as it is solely based on oxygenation, it has many drawbacks and poorly accounts for individual patient variability. In recent years, personalized approaches have gained attention, including the use of esophageal pressure monitoring [10], aiming for a slightly positive end-expiratory transpulmonary pressure (P_L_), and electrical impedance tomography (EIT) [11], targeting a compromise between lung overdistention and collapse (crossing point) [12]. The highest respiratory system compliance (C_RS_) is a classical individualized approach [13, 14], with the advantage that it could be determined without additional equipment other than the ventilator. It has been recently suggested that these approaches may result in very different levels of PEEP [15].

One primary goal for PEEP was to prevent alveolar collapse at the end of expiration and reverse severe hypoxemia [16]; however, it quickly became clear that PEEP could also result in alveolar overdistention and compromise hemodynamics, the latter resulting in a paradoxical benefit on oxygenation [17, 18]. Numerous experimental studies [5, 19–22], assessing the physiological effects of PEEP, have observed that both low and high levels of PEEP may be associated with ventilator-induced lung injury (VILI), and clinical trials have failed to prove a benefit from one approach versus the other on clinical outcomes [9, 23, 24]. A recent network metanalysis [25] has shown that the use of a higher PEEP (without prolonged lung recruitment strategy) was associated with lower risk of mortality than a lower PEEP strategy in patients with moderate to severe lung injury.

An often-overlooked aspect of PEEP titration is its impact on pulmonary hemodynamics, especially right ventricular afterload, and the interdependence between overall lung volume, blood gases and the overall resistance of pulmonary circulation. There is a classical relationship in physiology between pulmonary vascular resistance (PVR) and lung volumes, exhibiting a U-shape relationship, with the lowest PVR at the functional residual capacity (FRC) [26–28]. Based on this understanding, it has been assumed that the application of PEEP generally leads to a departure from FRC, ultimately resulting in an increase in PVR, as supported by earlier studies [29, 30]. A recent study [31] in 23 acute respiratory distress (ARDS) patients observed a negative impact of PEEP on PVR, especially in patients with low lung recruitability, indicated by recruitment-to-inflation ratio (R/I) < 0.5 (where PEEP is expected to create lung overdistention). In contrast, our previous experimental randomized trial suggested that pulmonary hemodynamics was commonly worse at low PEEP levels, especially when associated with massive lung collapse [5]. These contradictory findings raise concerns about our current PEEP titration strategies, highlighting the need to better understand the relationship between PEEP and pulmonary hemodynamics, especially during different degrees of lung injury—rather than in normal physiological conditions.

The main objectives of this study were 1) to estimate the PEEP level resulting from three different titration strategies, respectively using maximum C_RS_, EIT (crossing point), or P_L_ (slightly positive) in two porcine models of acute lung injury with different recruitability (bilateral vs. asymmetrical); and 2) to assess the impact of PEEP on pulmonary hemodynamics in acutely injured lungs and compare the impact of the different titration methods on pulmonary circulation.

## Methods

This is a secondary analysis of two series of experimental studies in two porcine models of acute lung injury [5, 32–34]. The study protocol was approved by the Animal Care Committee of the Peter Gilgan Centre for Research and Learning (PGRLC) at The Hospital for Sick Children – SickKids, reference number 1000058058. In this study, we included two series of experiments: I) Bilateral Lung Injury and II) Asymmetrical Lung Injury, as described in the following subsections.

Female Yorkshire pigs (35–50 kg) were sedated and paralyzed during the entire protocol. After intubation, pulmonary artery and femoral artery catheters (Swan-Ganz, Edwards Lifesciences, Irvine, United States, and PiCCO2, Getinge, Solna, Sweden), esophageal and gastric balloons (NutriVent®, Sidam, Italy), and EIT belt (PulmoVista® 500, Drager, Germany) were placed.

### Experiment series I: bilateral lung injury

In the first series of experiments, after animal preparation, we induced lung injury in both lungs by a two-hit model (surfactant lavage and high stretch ventilation) [5].

After inducing lung injury, we performed a decremental PEEP titration from PEEP of 24–22 cmH_2_O to 4–0 cmH_2_O, by steps of 2 cmH_2_O (≥ 30 s/step), either on volume-controlled ventilation (with tidal volume of 6 mL/kg), when the EIT device was connected to the ventilator, or on pressure-controlled ventilation (with driving pressure of 15 cmH_2_O). During the decremental PEEP titration, we continuously recorded airway pressure and flow, esophageal pressure, systemic blood pressure, central venous pressure (CVP), and pulmonary artery pressure (PAP), using PowerLab and LabChart (ADInstruments, Dunedin, New Zealand). At each step of PEEP, we calculated C_RS_—as tidal volume divided by (plateau pressure minus total PEEP), end-expiratory P_L_—as airway pressure minus esophageal pressure (both at end expiration), mean arterial pressure (MAP), mean PAP, and right-ventricular (RV) systolic transmural pressure—as systolic PAP minus expiratory esophageal pressure. EIT data were also recorded continuously, and we measured the percentage of lung collapse, lung overdistention, and regional ventilation at each level of PEEP. Airway opening pressure and R/I ratio were estimated [35].

Optimal PEEP was titrated using three different methods:

1. EIT: optimal PEEP was identified at the crossing point between collapse and overdistention.
2. End-expiratory P_L_: optimal PEEP was defined as the lowest PEEP level at which end-expiratory P_L_ was higher than zero.
3. C_RS_: optimal PEEP corresponded to the level that resulted in the highest C_RS_.

In two experiments of this series, we performed additional measurements to better understand the impact of PEEP and pulmonary hemodynamics on gas exchange and heart function. At each PEEP level, we recorded end-tidal carbon dioxide (EtCO_2_), collected arterial and mixed venous blood samples, and measured cardiac output (CO) and pulmonary capillary wedge pressure (PCWP). PVR was calculated as mean PAP minus PCWP divided by CO and multiplied by 80 [36].

### Experiment series II: asymmetrical lung injury

In the second series of experiments, we induced lung injury in only one lung [33]. After animal preparation, selective lung intubation of the left lung was performed with a double lumen endotracheal tube with confirmation via EIT. Surfactant lavage and high stretch ventilation were performed only on the left lung, while the right lung was collapsed. After inducing lung injury, bilateral ventilation was restored with a single lumen endotracheal tube. Decremental PEEP titration and variables measurements were performed as in the first series of experiments. In nine experiments of this series, we also performed measurements of CO, PCWP, and PVR.

### Data analysis and statistics

Categorical variables were expressed as percentage, and continuous variables were presented as mean and standard deviation or median and interquartile range according to their distribution. Statistical tests were two-tailed with α = 0.05, conducted using R (https://www.R-project.org/].

To compare the three levels of PEEP, we used repeated measures ANOVA and Duncan’s test. Correlations were assessed by Pearson’s test. Bland-Altman plots were built to estimate the mean difference between PEEP levels. Regression models were used to evaluate the relationship between PEEP and hemodynamic variables. To account for potential non-linear effects, we also explored quadratic regression models and evaluated the model fit using the Akaike information criterion (AIC). The dependent variable was pulmonary hemodynamics (i.e. mean PAP, RVTMP, or PVR), and the fixed effects included PEEP and PEEP squared. The intercept and the β coefficients for PEEP and PEEP squared were estimated, with standard errors (SE) provided for statistical inference. Linear mixed-effects model was employed to account for inter-case variability when analyzing the relationship between PEEP and mean PAP, and we calculated both the marginal and conditional R^2^ values. The marginal R^2^ represents the proportion of variance in the outcome variable explained by the fixed effects alone, while the conditional R^2^ reflects the variance explained by both the fixed effects and the random effects.

See extended methods in the supplements for more details.

## Results

### Experiment series I: bilateral lung injury

#### 1) PEEP levels and regional compliance

Thirty-seven pigs with bilateral lung injury were included in the first series of experiments. After lung injury, mean PaO_2_/FiO_2_ was 123 ± 74 mmHg and C_RS_ was 13 ± 3 mL/cmH_2_O at PEEP of 10 cmH_2_O. This model of lung injury was highly recruitable, with a median R/I ratio of 1.24(0.90–1.40).

**Fig. 1:**
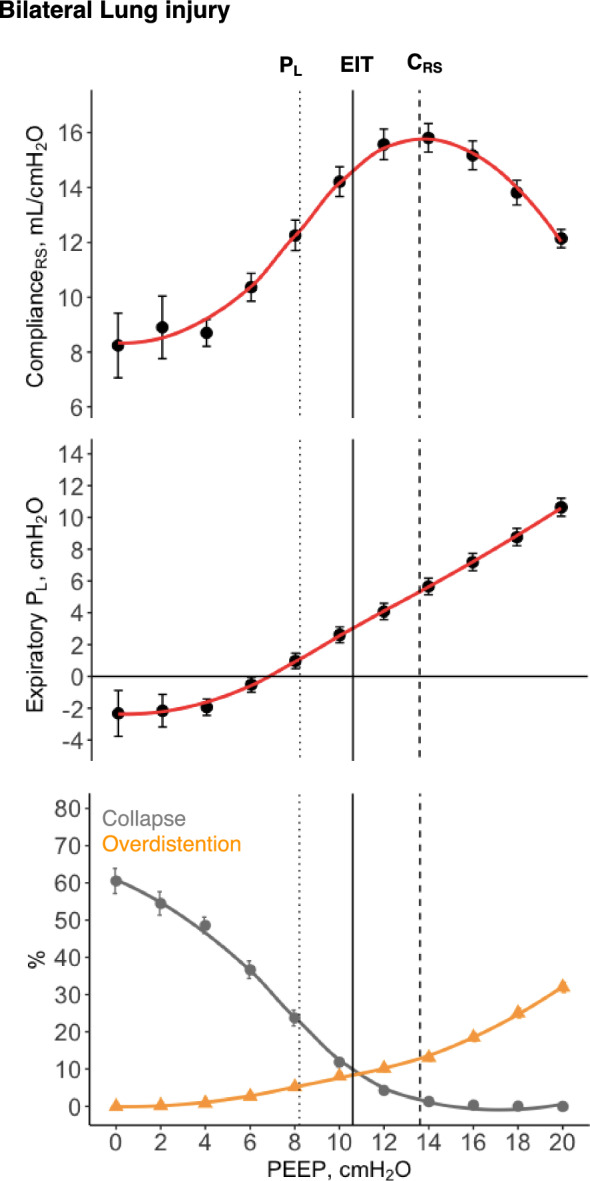
Positive end-expiratory pressure (PEEP) titration in pigs with bilateral lung injury (n = 37) according to three strategies. Black solid line represents the average electrical impedance tomography (EIT) crossing point between collapse and overdistension. Black dashed line represents the average highest respiratory system compliance (C_RS_). Black dotted line represents the average end-expiratory transpulmonary pressure (P_L_) slightly positive. Error bars represent standard error

**Fig. 2:**
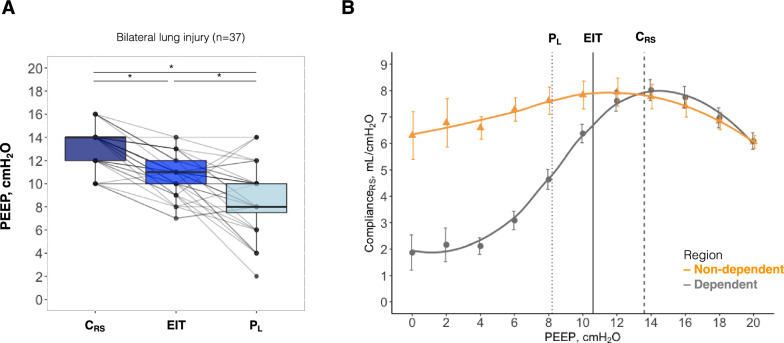
**A** The optimal positive end-expiratory pressure (PEEP) according to different PEEP titration strategies in pigs with bilateral lung injury (n = 37): C_RS_, highest respiratory system compliance; EIT, electrical impedance tomography crossing point; and P_L_, end-expiratory transpulmonary pressure slightly positive. * represents *post-hoc* *p* < 0.05. **B** Regional compliance in bilateral lung injury (n = 37). Black solid line represents the average EIT crossing point between collapse and overdistension. Black dashed line represents the average highest C_RS_. Black dotted line represents the average end-expiratory P_L_ slightly positive. Error bars represent standard error

The three strategies of PEEP titration performed in this study resulted in three different levels of optimal PEEP in this model (Fig. 1): median PEEP was 14(12–14) cmH_2_O at the highest C_RS_, 11(10–12) cmH_2_O at the EIT crossing point, and 8(8–10) cmH_2_O at slightly positive expiratory P_L_, *p* < 0.001 (Fig. 2A).

There was a moderate correlation between C_RS_-PEEP and EIT-PEEP (Pearson's r = 0.59, *p* < 0.001), with a mean difference of 2 cmH_2_O (Supplemental Fig. 1A), and a weak, non-significant, correlation between C_RS_-PEEP and P_L_-PEEP (Pearson's r = 0.16, *p* = 0.345), with a mean difference of 4 cmH_2_O (Supplemental Fig. 1B).

In terms of regional respiratory system compliance (Fig. 2B), PEEP set based on C_RS_ was almost a perfect reflection of the compliance of the dependent lung. The mean difference between ventral and dorsal ventilation was higher at PEEP set based on end-expiratory P_L_ (18 ± 31%), than for both PEEP based on EIT (3 ± 19%, *p* = 0.008 and on C_RS_ (0 ± 18%, *p* = 0.002), Supplemental Fig. 2.

#### 2) Impact on pulmonary hemodynamics

**Fig. 3:**
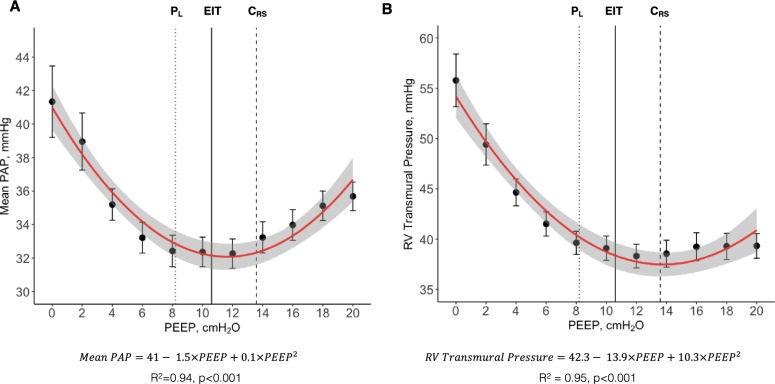
Pulmonary hemodynamics in bilateral lung injury (n = 37):** A** Mean pulmonary artery pressure (Mean PAP) at each level of positive end-expiratory pressure (PEEP). **B** Right ventricular (RV) systolic transmural pressure at each level of PEEP. Dots represent mean and error bars represent standard error. Red line represents quadratic regression, and grey area shaded represents 95% confidence interval. Black solid line represents the average electrical impedance tomography (EIT) crossing point between collapse and overdistension. Black dashed line represents the average highest respiratory system compliance (C_RS_). Black dotted line represents the average end-expiratory transpulmonary pressure (P_L_) slightly positive

We observed a quadratic relationship (U shape) between mean PAP and PEEP (Fig. 3A). The quadratic model (AIC = 29.7) provided a better fit to the data compared to the linear model (AIC = 56.6), as indicated by the lower AIC value. Similarly, the relationship between RV systolic transmural pressure and PEEP was also quadratic (Fig. 3B). To further investigate these relationships while accounting for inter-case variability, we performed a linear mixed-effects model, including a quadratic term, which confirmed the quadratic relationship (*p* < 0.001). The conditional R^2^ was 0.905, indicating that 90.5% of the variance in mean PAP can be explained by the model, while the marginal R^2^ was 0.041, reflecting the variance explained by the fixed effects alone. The β coefficients for PEEP and PEEP squared were − 1.18 (SE = 0.070, *p* < 0.001) and 0.053 (SE = 0.003, *p* < 0.001), respectively, with a random effect variance of 56.15 for the intercept and a residual variance of 6.21. These results demonstrate that mean PAP decreases with increasing PEEP up to a certain point, after which it begins to increase again, supporting the hypothesis of a quadratic (non-linear) relationship. We observed a negative linear correlation of mean systemic arterial pressure (conditional R^2^:0.88, *p* < 0.001) and CO (conditional R^2^:0.96, *p* < 0.001) with PEEP, a positive linear correlation between CVP and PEEP (conditional R^2^ = 0.97, *p* < 0.001), and a quadratic relationship between transmural CVP and PEEP (conditional R^2^ = 0.81) Supplemental Fig. 3.

### Experiment series II: asymmetrical lung injury

#### 1) PEEP levels and regional compliance

**Fig. 4:**
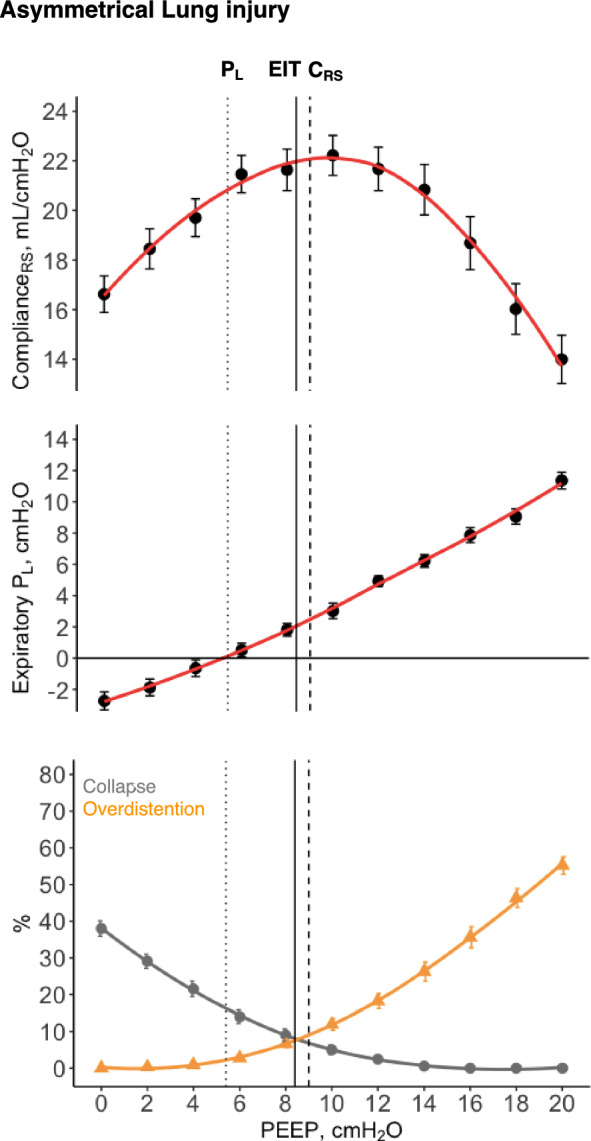
Positive end-expiratory pressure (PEEP) titration in pigs with asymmetrical lung injury (n = 13) according to three strategies. Black solid line represents the average electrical impedance tomography (EIT) crossing point between collapse and overdistension. Black dashed line represents the average highest respiratory system compliance (C_RS_). Black dotted line represents the average end-expiratory transpulmonary pressure (P_L_) slightly positive. Error bars represent standard error

**Fig. 5:**
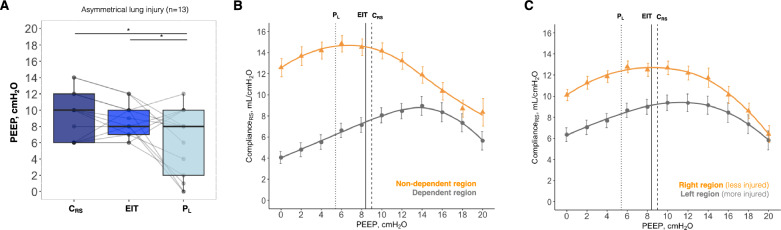
Optimal PEEP and regional respiratory system mechanics in asymmetrical lung injury (n = 13). **A** The optimal positive end-expiratory pressure (PEEP) according to different PEEP titration strategies in pigs with asymmetrical lung injury: C_RS_, highest respiratory system compliance; EIT, electrical impedance tomography crossing point; and P_L_, end-expiratory transpulmonary pressure slightly positive. * represents *post-hoc* *p* < 0.05. **B** Dependent (grey) versus non-dependent (orange) regions. **C** Left (grey) versus right (orange) regions. Surfactant lavage and high-stretch ventilation were performed in the left side (more injured), while the right side was kept collapsed (less injured). Black solid line represents the average EIT crossing point between collapse and overdistension. Black dashed line represents the average highest C_RS_. Black dotted line represents the average end-expiratory P_L_ slightly positive. Error bars represent standard error

Thirteen pigs with asymmetrical lung injury were included in the second series of experiments. After lung injury, mean PaO_2_/FiO_2_ was 172 ± 48 mmHg and total C_RS_ was 22 ± 4 mL/cm H_2_O at PEEP of 10 cmH_2_O. This model of lung injury had low-to-moderate lung recruitability, with a median R/I ratio of 0.67(0.29–0.98). The PEEP at slightly positive expiratory P_L_ (6 ± 4 cmH_2_O) was lower than both PEEP at the EIT crossing point (9 ± 2 cmH_2_O, *p* = 0.047) and PEEP at the highest C_RS_ (9 ± 3 cmH_2_O, *p* = 0.023), despite a noteworthy variability of distribution (Figs. 4 and 5A). There was a strong correlation between C_RS_-PEEP and EIT-PEEP (Pearson's r = 0.78, *p* = 0.002), with a mean difference of 0 cmH_2_O, and a weak, non-significant, correlation between C_RS_-PEEP and P_L_-PEEP (Pearson's r = 0.32, *p* = 0.282), with a mean difference of 4 cmH_2_O (Supplemental Fig. 4).

Regarding regional respiratory mechanics in asymmetrical lung injury, right versus left regional C_RS_ and dependent versus non-dependent regional C_RS_ were not significantly different across the three approaches (Fig. 5). The mean absolute difference between ventral and dorsal regional ventilation tended to be higher at PEEP based on end-expiratory P_L_ (38 ± 21%), than at PEEP based on EIT (27 ± 24%, *p* = 0.080) and at PEEP based on total C_RS_ (26 ± 25%, *p* = 0.080), Supplemental Fig. 5. The mean absolute difference between right and left percentage of ventilation was 20 ± 15% at PEEP based on end-expiratory P_L_, 16 ± 12% at PEEP based on EIT, and 14 ± 11% at PEEP based on total C_RS_, *p* = 0.568.

#### 2) Impact on pulmonary hemodynamics

**Fig. 6:**
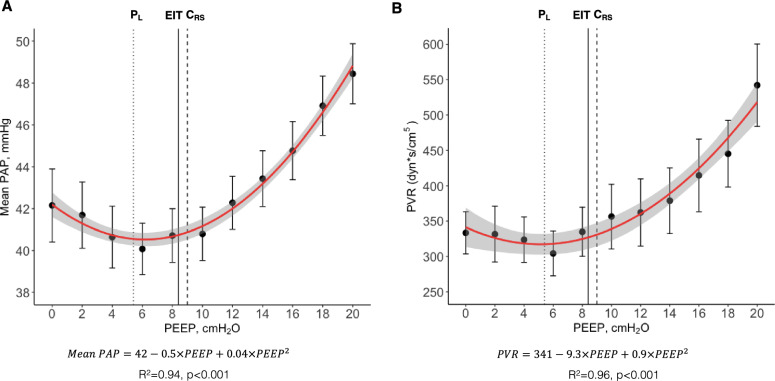
Pulmonary hemodynamics in asymmetrical lung injury (n = 13):** A** Mean pulmonary artery pressure (Mean PAP) at each level of positive end-expiratory pressure (PEEP). **B** Pulmonary vascular resistance (PVR) at each level of PEEP. Dots represent mean and error bars represent standard error. Red line represents quadratic regression, and grey shaded area represents 95% confidence interval. Black solid line represents the average electrical impedance tomography (EIT) crossing point between collapse and overdistension. Black dashed line represents the average highest respiratory system compliance (C_RS_). Black dotted line represents the average end-expiratory transpulmonary pressure (P_L_) slightly positive

In pigs with asymmetrical lung injury, a quadratic relationship was also found between PEEP and mean PAP (Fig. 6A) and PEEP and RV systolic transmural pressure (y = 47.0–0.14x + 7.78x^2^; R^2^ = 0.93, *p* < 0.001). The quadratic model for mean PAP and PEEP (AIC = 11.8) also provided a better fit compared to the linear model (AIC = 47.0). The conditional R^2^ was 0.811, indicating that 81.1% of the variance in mean PAP can be explained by the model, while the marginal R^2^ was 0.113, reflecting the variance explained by the fixed effects alone, respectively, with a random effect variance of 40.73 for the intercept and a residual variance of 11.01. The β coefficients for PEEP and PEEP squared in the mixed model analysis were − 0.54 (SE = 0.116, *p* < 0.001) and 0.044 (SE = 0.006, *p* < 0.001). In nine pigs of this series of experiments, we measured PVR to verify if the response in mean PAP to PEEP would represent the response in PVR, and we found that PVR behaved like mean PAP (Fig. 6B). Systemic hemodynamics behaved similarly to pigs with bilateral lung injury (Supplemental Fig. 6).

### Sensitivity analysis

**Fig. 7:**
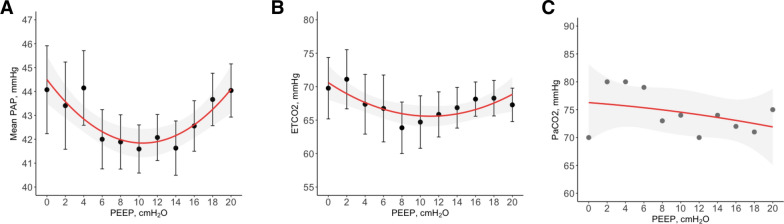
Sensitivity analysis in 5 animals in volume-controlled ventilation. **A** Mean pulmonary artery pressure (Mean PAP) at each level of positive end-expiratory pressure (PEEP), n = 5. **B** End-tidal carbon dioxide (EtCO_2_) at each level of PEEP, n = 5. **C** Partial pressure of arterial carbon dioxide (PaCO_2_) at each level of PEEP measured in one of the experiments. Error bars represent standard error. Grey shaded area represents 95% confidence interval

To assess whether changes in PaCO_2_ were responsible for the observed variability in PVR, we conducted a sensitivity analysis using data from PEEP titrations performed in volume-controlled ventilation (keeping minute ventilation constant), where CO_2_ levels remained stable. The results of this analysis were consistent with the original findings. In this small subgroup (n = 5), the quadratic relationship between PEEP and mean PAP was also present, with a β coefficients for PEEP of − 0.51 (SE = 0.177, *p* = 0.005) and for PEEP squared of 0.024 (SE = 0.009, *p* = 0.006). (Fig. 7A). When adjusting the model by EtCO_2_ and subject in a mixed model, the effect of EtCO_2_ was not significant (β coefficient = − 0.023, SE = 0.046, *p* = 0.617), while PEEP still had a quadratic relationship with mean PAP (β coefficient for PEEP of − 0.62 (SE = 0.20, *p* = 0.003) and for PEEP squared of 0.027 (SE = 0.0099, *p* = 0.006). Additionally, PaCO_2_ was measured at each PEEP level in one experiment, showing stable values (70–80 mm Hg) without a quadratic relationship (R^2^ = 0.15, *p* = 0.524). In animals submitted to PEEP titration on pressure-controlled ventilation, when assessing the relationship between PEEP and mean PAP, adjusted by EtCO_2_ and accounting for inter-case variability, EtCO_2_ also influenced mean PAP (Supplemental Fig. 7), with a β coefficient of 0.236 (SE = 0.022, *p* < 0.001), indicating that higher EtCO_2_ levels are associated with increased mean PAP.

## Discussion

In porcine models of bilateral and highly recruitable or asymmetrical and less recruitable acute lung injury, we found that: 1) PEEP titration based on EIT, C_RS_, and end-expiratory P_L_, resulted in three different levels of optimal PEEP in bilateral lung injury; 2) differences in optimal PEEP were smaller but still significant in asymmetrical lung injury; 3) PEEP had a quadratic relationship with mean PAP (i.e., high mean PAP at low and high levels of PEEP), RV systolic transmural pressure, and PVR; and 4) PEEP set at the EIT crossing-point or highest C_RS_ resulted in more homogeneous regional ventilation than at slightly positive end-expiratory P_L_ in bilateral lung injury.

In our study we measured the “optimal PEEP” based on three different approaches: at the EIT-crossing point, at slightly positive end-expiratory P_L_, and at the highest C_RS_. Similarly to previous studies [15, 37, 38], we observed that the three strategies resulted in three different levels of PEEP in our model of bilateral lung injury. Possible explanations for this finding are the different principles and limitations of each strategy and the relative impact of the different parts of the lungs and their distension. Setting PEEP either at the highest C_RS_ or at slightly positive end-expiratory P_L_ aims to achieve the maximal recruitment of the lungs and prevent atelectasis, based on respiratory mechanics [39]. However, global C_RS_ measured by the ventilator is mostly reflecting the dependent part of the lung in bilateral injury. In addition P_L_ provides information about the distending pressure of the lungs more specifically at the level of the esophagus [10, 40–43]. The EIT, on the other hand, accepts some level of collapse in order to achieve the best compromise between collapse and overdistention [11], estimated by changes in pixel compliance [44].

In the series of experiments with asymmetrical lung injury, we observed low to moderate recruitability, while the bilateral model was highly recruitable [35]. This could explain the lower mean optimal PEEP for all approaches, and a more pronounced increase in overdistention and decrease in C_RS_ while increasing PEEP in the asymmetrical lung injury. Our findings reinforce that the variability in PEEP levels recommended by different approaches extended beyond the global respiratory mechanics, and it highlights the need for a comprehensive understanding of how PEEP impacts other critical physiological aspects, such as regional lung mechanics and pulmonary vascular responses.

For decades, it was widely believed that any increase in PEEP would invariably lead to an increase in PVR due to the expected physiological increase in end-expiratory lung volume, based on the landmark work of Simmons *et. al.,* in 1961 [26], showing that PVR increases with either decrease or increase of lung volume from FRC. In our study, we observed a quadratic relationship (U shape) between PEEP and mean PAP, RV systolic transmural pressure, and PVR. These results support recent evidence [31] that PVR only decreases when PEEP results in lung recruitment. This may help explain previous evidence [45] that ARDS patients have different PVR responses to PEEP. The impairment in pulmonary hemodynamics at high PEEP may be explained by the effect of lung overdistention [26, 27, 46–48], compressing the intra-alveolar vessels, while the impairment at low PEEP may be caused not only by stretching extra-alveolar vessels, but also by atelectasis-induced vascular leaking, capillary folding, and hypoxic vasoconstriction [5, 20, 21, 49, 50], as represented in Supplemental Fig. 8. This would explain why we observed a higher increase in mean PAP at low levels of PEEP in pigs with bilateral lung injury (high levels of collapse at low PEEP, recruitable model) and higher increase in mean PAP at high levels of PEEP in pigs with asymmetrical lung injury (high levels of overdistention at high PEEP, poorly recruitable model). To support our hypothesis with EIT the percentage of collapse increased to an average of 60% at low PEEP and overdistention to 30% at high PEEP in the bilateral injury, while overdistention rose to 70% at high PEEP and collapse to 40% at low PEEP in the asymmetrical injury. These findings may explain the lower range of cardiac output and higher CVP observed in the asymmetrical model.

Our results suggest that in mechanically ventilated patients with acute lung injury there might be an optimal range of PEEP for the pulmonary hemodynamics, that results in an end-expiratory lung volume optimal for PVR. This hypothesis has also been suggested by Schmitt *et. al*. in 2001 [51], based on pulmonary artery Doppler flow velocity measurements in ARDS, comparing three levels of PEEP. All three PEEP selection approaches used in our study tended to recommend a level of PEEP within the lower levels of mean PAP in a decremental PEEP titration, especially EIT. Although the three approaches are grounded in different principles, they all focus on individualizing treatment based on patient-specific respiratory mechanics. This patient-centered strategy ensures that PEEP levels remain within a safe range of respiratory system mechanics, thereby creating favorable conditions for pulmonary hemodynamics.

In terms of regional mechanics, PEEP set based on the EIT and C_RS_ resulted in relative homogenization of regional ventilation in bilateral lung injury, as expected based on their individualized approach. PEEP set at slightly positive end-expiratory P_L_ tended to result in a higher difference between dorsal and regional ventilation. This disparity could be attributed to the fact that esophageal pressure primarily reflects pleural pressure in lung regions adjacent to the esophageal balloon (dependent and middle areas) [52]. As a result, significant variations in regional pleural or transpulmonary pressures might be either overestimated or underestimated when using the direct method to estimate P_L_ [39–41], as in our study. Our results highlight that a higher end-expiratory P_L_ should be targeted to keep the lung open (at least 2 cmH_2_O instead of at least 0 cmH_2_O), as hypothesized by previous study [53]. In the asymmetrical lung injury, the differences in regional C_RS_ at the optimal PEEP were high regardless of the method of PEEP titration, while in the bilateral injury they were sometimes close to zero (based on EIT and C_RS_). The regional C_RS_ in the non-dependent region of the lung was higher than in the dependent region, even at higher PEEP. This may be explained by the heterogeneity of injury in the asymmetrical model [32, 33], as only one lung was submitted to surfactant lavage and high stretch (more injured), while the other one was kept collapsed (less injured). This finding is supported by the fact that the regional C_RS_ in the lung that was kept collapsed (right) was always higher than in the other lung (left).

Our study has several limitations. First, one could argue that the two models of lung injury used in this study have underlying differences. However, our primary objective was to compare the impact of PEEP on pulmonary hemodynamics across different lung injury models, rather than to compare the models themselves. The use of various lung injury models was intended to represent different clinical scenarios and provide a comprehensive understanding of the mechanisms involved. Our study design and analysis focused on intra-model comparisons to reduce these potential confounding factors. Secondly, we did not measure PVR for all experiments. However, mean PAP is an adequate surrogate, and we observed a similar relationship between PVR and PEEP in the animals with these measurements. Measuring PVR and cardiac output considerably lengthened the PEEP titration process (with a risk of instability of the model over time), which is the reason that we measured PAP only in most experiments. Thirdly, most PEEP titrations were conducted in pressure-controlled ventilation (varying tidal volume), with consequent variability of PaCO_2_, which could also influence PVR. To determine if PaCO_2_ variability drove changes in PVR, we performed a sensitivity analysis using data from volume-controlled ventilation experiments, where CO_2_ levels were more stable, and the results were consistent. Moreover, it is important to note that the sensitivity analysis was conducted only in a small subsample, which may have influenced the results. Future research could focus on the PaCO_2_ impact on PVR. Fourthly, it's important to note that all the PEEP titration strategies examined were individualized approaches based on some aspect of global respiratory mechanics. Therefore, we cannot extrapolate our findings to methods based on other variables, such as oxygenation. Finally, we acknowledge that critical illness adds significant complexity to the interaction between PEEP and hemodynamics. Individualized PEEP titration may optimize pulmonary hemodynamics, while lower PEEP levels could offer immediate advantages for systemic hemodynamics. However, these advantages are short-term results, and it is unlikely that keeping a lower PEEP while exposing the right ventricle to a higher afterload can ultimately be beneficial. If the right ventricular function worsens the left ventricle will follow what is supplied by the right side. Our findings provide a foundational understanding of these dynamics and clinicians must also account for factors such as pre-existing cardiovascular dysfunction, fluid balance, and the effects of vasoactive drugs when translating these results into clinical practice.

## Conclusions

In porcine models of acute lung injury (highly and poorly recruitable), both very low and very high levels of PEEP may impair pulmonary hemodynamics, increasing mean PAP and potentially PVR either by significantly increasing lung collapse or by causing lung overdistention. The relationship between mean PAP and PEEP follows a “U shape”, with the best range observed at levels of PEEP individualized based on respiratory mechanics, especially by EIT and esophageal pressure monitoring.

## Supplementary Information

Additional file1 (DOCX 2158 KB)

## Data Availability

Data is provided within the manuscript or supplementary information files.
