# Supplementary material for: Individualized PEEP can improve both pulmonary hemodynamics and lung function in acute lung injury
Source: Crit Care. 2025 Mar 10;29:107. doi: 10.1186/s13054-025-05325-7 (PMC11892255; doi:10.1186/s13054-025-05325-7)
Supplement: Supplementary file 1 — Additional file1 (DOCX 2158 KB) [file 13054_2025_5325_MOESM1_ESM.docx]

**Individualized PEEP can Improve both Pulmonary Hemodynamics and Lung Function in Acute Lung Injury**

Mayson L. A. Sousa, Luca S. Menga, Annia Schreiber, Mattia Docci, Fernando Vieira, Bhushan H. Katira, Mariangela Pellegrini, Sebastian Dubo, Ghislaine Douflé, Eduardo L. V. Costa, Martin Post, Marcelo B. P. Amato, Laurent Brochard

**Supplemental Material**

**Extended Methods**

Female Yorkshire pigs (35-50kg) were sedated with Pentobarbital and paralyzed with Rocuronium during the entire protocol. After endotracheal intubation, a pulmonary artery catheter (Swan-Ganz, Edwards Lifesciences, Irvine, United States) was inserted in the right external jugular vein and an arterial catheter (PiCCO2, Getinge, Solna, Sweden) was inserted in the femoral artery, for systemic and pulmonary hemodynamics monitoring and blood sampling. An esophageal and gastric balloon catheter (NutriVent^®^, Sidam, Italy) was placed in the esophagus. An EIT belt, PulmoVista^®^ 500 (Drager, Germany), was placed around the thorax, just below the front legs. We performed the same animal preparation for both series of experiments.

*Experiment Series I: Bilateral Lung Injury*

In the first series of experiments, after animal preparation, we induced lung injury in both lungs by a two-hit model: 1) surfactant lavage until PaO_2_/FiO_2_<100mmHg and 2) high stretch ventilation for up to 2 hours, as previously described(1).

After inducing lung injury, we performed a decremental PEEP titration from PEEP of 24-22 cmH_2_O to 4-0 cmH_2_O, by steps of 2 cmH_2_O (at least 30 seconds each, according to hemodynamic stability), either on volume-controlled ventilation (with tidal volume of 6 mL/kg), when the EIT device was connected to the ventilator, or on pressure-controlled ventilation (with driving pressure of 15 cmH_2_O). Respiratory rate was set at 20-25 breaths/min. During the decremental PEEP titration we continuously recorded airway pressure and flow, esophageal pressure, systemic blood pressure, central venous pressure (CVP), and pulmonary artery pressure (PAP), using PowerLab and LabChart (ADInstruments, Dunedin, New Zealand). At each step of PEEP, we calculated C_RS_ – as tidal volume divided by plateau pressure minus total PEEP, end-expiratory P_L_ – as airway pressure minus esophageal pressure (both at end expiration), mean arterial pressure (MAP), mean PAP, and right-ventricle (RV) systolic transmural pressure – as systolic PAP minus expiratory esophageal pressure. EIT data were also recorded continuously with a sample rate of 50 Hz, and we measured the percentage of lung collapse, lung overdistention, and regional ventilation at each level of PEEP. Regional C_RS_ was estimated as regional percentage of ventilation (right, left, dependent, and non-dependent) times global C_RS_. A low-flow inflation maneuver (5L/min) from PEEP of 0 cmH_2_O was performed to measure the airway opening pressure followed by a single breath maneuver from PEEP of 15 to 5 cmH_2_O to measure the R/I ratio, as previously described(2).

Optimal PEEP was titrated by three different methods:

1. EIT: optimal PEEP was identified at the crossing point between collapse and overdistention.
2. End-expiratory P_L_: optimal PEEP was defined as the lowest PEEP level at which end-expiratory P_L_ was higher than zero.
3. C_RS_: optimal PEEP corresponded to the level that resulted in the highest C_RS_.

In two experiments of this series, we performed additional measurements to better understand the impact of PEEP and pulmonary hemodynamics on gas exchange and heart function. At each PEEP level, we recorded end-tidal carbon dioxide (EtCO_2_) using a mainstream capnometer (Infinity^®^ MCable^TM^- Drager, Germany), collected arterial and mixed venous blood samples, and measured cardiac output (CO) and pulmonary capillary wedge pressure (PCWP). CO was measured twice at each time point, and we recorded the average value. PVR was calculated as mean PAP minus PCWP divided by CO and multiplied by 80(3).

*Experiment Series II: Asymmetrical Lung Injury*

In the second series of experiments, we induced lung injury in only one lung, as previously described(4). After animal preparation, selective lung intubation of the left lung was performed with a double lumen endotracheal tube with confirmation via EIT. Surfactant lavage until PaO_2_/FiO_2_<100mmHg and high stretch ventilation for 30 minutes was performed only on the left lung, while the right lung was collapsed. After inducing lung injury, bilateral ventilation was restored with a single lumen endotracheal tube. Decremental PEEP titration and variables measurements (C_RS_, end-expiratory P_L_, MAP, CVP, mean PAP, EIT) were performed as in the first series of experiments. In nine experiments of this series, we also performed measurements of CO, PCWP, and PVR.

*Data Analysis and Statistics*

Categorical variables were expressed as percentage, and continuous variables were presented as mean and standard deviation or median and interquartile range according to their distribution, assessed by histogram and Shapiro Wilk-Test. Statistical tests were two-tailed with type-I error (alpha) set at 0.05. The statistical analyses were performed using R software (<https://www.R-project.org/>).

To compare the three levels of PEEP we used repeated measures ANOVA and Duncan’s test. The correlation between the optimal PEEP levels titrated by different methods was assessed using Pearson’s test. Bland-Altman plots were built to estimate the mean difference between PEEP levels. Regression models were used to evaluate the relationship between PEEP and hemodynamic variables. To account for potential non-linear effects, we also explored quadratic regression models and evaluated the model fit using the Akaike information criterion (AIC). The dependent variable was pulmonary hemodynamics (i.e. mean PAP, RVTMP, or PVR), and the fixed effects included PEEP and PEEP squared. The intercept and the β coefficients for PEEP and PEEP squared were estimated, with standard errors (SE) provided for statistical inference. Linear mixed-effects model was employed to account for inter-case variability when analyzing the relationship between PEEP and mean PAP, and we calculated both the marginal and conditional R² values. The marginal R² represents the proportion of variance in the outcome variable explained by the fixed effects alone, while the conditional R² reflects the variance explained by both the fixed effects and the random effects. A sensitivity analysis was performed to assess the impact of PaCO2 on the relationship between PEEP and mean PAP. Animals were regrouped according to the ventilation mode, pressure-controlled ventilation versus volume-controlled ventilation, with lower variability of PaCO_2_ in the second one, and mixed-effects models were adjusted by EtCO_2_.

**Supplementary Results**

**Supplemental Figure 1. *Bilateral Lung Injury (n=36):* A)** Relationship between positive end-expiratory pressure (PEEP) titrated by respiratory system compliance (C_RS_) and by electrical impedance tomography (EIT). **B)** Relationship between PEEP) titrated by C_RS_ and end-expiratory transpulmonary pressure (Expiratory P_L_). Grey shaded area represents regression line 95% confidence interval. Blue dashed line represents mean difference. Red dotted line represents standard deviation.

**Supplemental** **Figure 2.** Regional ventilation in bilateral lung injury (n=37). A) Difference between ventral (non-dependent region of the lung) and dorsal (dependent region of the lung) ventilation across the three strategies of positive-end-expiratory pressure (PEEP) titration. B) Representative ventilation maps. C_RS_, highest respiratory system compliance; EIT, electrical impedance tomography crossing point; and P_L_, end-expiratory transpulmonary pressure slightly positive. * represents *post-hoc* p<0.05.

**Supplemental** **Figure 3.** Systemic hemodynamics in bilateral lung injury (n=37*): **A)** Mean systemic arterial pressure at each level of positive end-expiratory pressure (PEEP). **B)** Central venous pressure (CVP) at each level of PEEP. **C)** Transmural CVP pressure at each level of PEEP. **D)** Cardiac output at each level of PEEP*. Dots represent mean and error bars represent standard error. Red line represents linear regression, and grey shaded area represents 95% confidence interval. Black solid line represents the average electrical impedance tomography (EIT) crossing point between collapse and overdistension. Black dashed line represents the average highest respiratory system compliance (C_RS_). Black dotted line represents the average end-expiratory transpulmonary pressure (P_L_) slightly positive. Error bars represent standard error. *Cardiac output was measured in only 2 experiments in this series.

**Supplemental Figure 4. *Asymmetrical Lung Injury* (n=13): A)** Relationship between positive end-expiratory pressure (PEEP) titrated by respiratory system compliance (C_RS_) and by electrical impedance tomography (EIT). **B)** Relationship between PEEP) titrated by C_RS_ and end-expiratory transpulmonary pressure (Expiratory P_L_). Grey shaded area represents regression line 95% confidence interval. Blue dashed line represents mean difference. Red dotted line represents standard deviation.

**Supplemental** **Figure 5.** Regional ventilation in asymmetrical lung injury (n=13). A) Difference between ventral (non-dependent region of the lung) and dorsal (dependent region of the lung) ventilation across the three strategies of positive-end-expiratory pressure (PEEP) titration. B) Representative ventilation maps. C_RS_, highest respiratory system compliance; EIT, electrical impedance tomography crossing point; and P_L_, end-expiratory transpulmonary pressure slightly positive.

**Supplemental** **Figure 6.** Systemic hemodynamics in asymmetrical lung injury (n=13*): **A)** Mean systemic arterial pressure at each level of positive end-expiratory pressure (PEEP). **B)** Central venous pressure at each level of PEEP. Dots represent mean and error bars represent standard error. **C)** Transmural CVP pressure at each level of PEEP. **D)** Cardiac output at each level of PEEP*. Red line represents linear regression, and grey shaded area represents 95% confidence interval. Black solid line represents the average electrical impedance tomography (EIT) crossing point between collapse and overdistension. Black dashed line represents the average highest respiratory system compliance (C_RS_). Black dotted line represents the average end-expiratory transpulmonary pressure (P_L_) slightly positive. Error bars represent standard error. * For cardiac output, n=9.


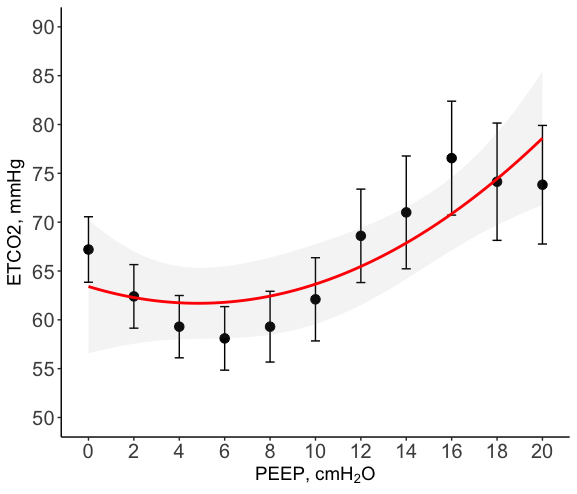


**Supplemental** **Figure 7.** End-tidal carbon dioxide (EtCO2) at each level of positive end-expiratory pressure (PEEP) during a decremental PEEP titration in five animals on pressure-controlled ventilation.

**Supplemental** **Figure 8.** Possible explanation for the U shape curve relating positive end-expiratory pressure (PEEP) and pulmonary vascular resistance (PVR). There might be an optimal level of PEEP where both intra-alveolar and extra-alveolar vessels have low resistance, similar to functional residual capacity (FRC).

**References**

1. Sousa MLA, Katira BH, Bouch S, Hsing V, Engelberts D, Amato M, *et al.* Limiting Overdistention or Collapse when Mechanically Ventilating Injured Lungs: A Randomized Study in a Porcine Model. *Am J Respir Crit Care Med* 2024;doi:10.1164/rccm.202310-1895OC.

2. Chen L, Del Sorbo L, Grieco DL, Junhasavasdikul D, Rittayamai N, Soliman I, *et al.* Potential for Lung Recruitment Estimated by the Recruitment-to-Inflation Ratio in Acute Respiratory Distress Syndrome. A Clinical Trial. *Am J Respir Crit Care Med* 2020;201:178–187.

3. Kwan WC, Shavelle DM, Laughrun DR. Pulmonary vascular resistance index: Getting the units right and why it matters. *Clin Cardiol* 2019;42:334–338.

4. Bastia L, Engelberts D, Osada K, Katira BH, Damiani LF, Yoshida T, *et al.* Role of Positive End-Expiratory Pressure and Regional Transpulmonary Pressure in Asymmetrical Lung Injury. *Am J Respir Crit Care Med* 2021;203:969–976.
